# A novel method to extend viability and functionality of living heart slices

**DOI:** 10.3389/fcvm.2023.1244630

**Published:** 2023-10-10

**Authors:** Abigail J. Ross, Iva Krumova, Berfin Tunc, Qin Wu, Changhao Wu, Patrizia Camelliti

**Affiliations:** ^1^ School of Biosciences and Medicine, University of Surrey, Guildford, United Kingdom; ^2^ School of Medicine, Jiangsu Vocational College of Medicine, Yancheng, China

**Keywords:** myocardial slices, organotypic *ex-vivo* models, BDM (2,3-Butanedione monoxime), myosin II ATPase, contractility, field potential, porcine, 3Rs

## Abstract

Living heart slices have recently emerged as a powerful experimental model for fundamental cardiac research. By retaining the structure and function of the native myocardium while maintaining the simplicity of cell culture models, heart slices can be easily employed in electrophysiological, pharmacological, biochemical, and structural investigations. One single heart yields many slices (>20 slices for rodents, >100 slices for porcine or human hearts), however due to the low throughput of most assays and rapid slice degeneration within 24 h of preparation, many slices remain unused and are discarded at the end of the preparation day. Here we present a novel method to extend viability and functionality of living heart slices, enabling their use in experiments over several consecutive days following preparation. By combining hypothermic conditions with inhibition of myosin II ATPase using 2,3-butanedione monoxime (BDM), slices prepared from the left ventricle of porcine hearts remain viable and exhibit preserved contractile function and morphology for up to 6 days. Electrophysiological function was also confirmed over the 6 days by extracellular field potentials recordings. This simple method not only maximizes the use of slices prepared from one single heart, thus reducing the number of animals required, but also increases data reproducibility by allowing multiple electrophysiological, pharmacological, biochemical, and structural studies to be performed from the same heart.

## 1. Introduction

Living heart tissue slices have recently gained momentum as a powerful tool in cardiac research. Ultrathin sections prepared from living ventricular myocardium, heart slices retain native cardiac tissue multicellularity, extracellular matrix, cell-cell contacts, electrophysiology, and contractility, while maintaining the simplicity of an isolated, controlled environment system (1, 2). Slices obtained from the heart of rodents and large mammals, including human, have been employed in acute electrophysiological, pharmacological, biochemical, and structural investigations. Applications include the study of functional regional and transmural heterogeneities, remodelling occurring with heart disease and mechanisms of cardiac regeneration (3–8). Acute heart slices have also been identified as a powerful platform for cardiotoxicity studies (2, 9–12).

In general, preparation of slices requires sacrificing an animal, but one single heart yields many slices (>20 slices for rat heart, >100 slices for pig heart). However, the low-throughput nature of most assays employed to assess electrophysiology and mechanics limits the number of slices that can be studied on the day of preparation. Many slices that could be used for acute experiments remain unused and are discarded at the end of the preparation day. Using currently available protocols, slices can be successfully stored for 12–24 h with no significant electrophysiological changes (2, 13). Extending this window to several days will allow to maximize the use of slices prepared from one single heart. Slices from the same heart could be used over consecutive days, accelerating cardiac research, and decreasing the number of animals needed for experiments. For human slices, fresh tissue obtained during cardiac surgeries or unused donor hearts are needed. These samples are extremely limited and are only available to a small number of research groups. In this context a robust and simple protocol to preserve slices would prevent waste of valuable material and could further promote sharing of living slices between laboratories.

Combining low temperature with inhibition of myosin II ATPase has been previously shown to preserve viability and functionality of isolated cardiomyocytes. Canine cardiomyocytes stored at 4°C in physiological solution containing the myosin II ATPase inhibitor N-benzyl-p-toluene sulphonamide (BTS) maintain morphology, action potential, calcium cycling and sarcomere shortening comparable to freshly isolated cells for 1 week after cell isolation (14). Rat cardiomyocytes stored at 4°C in physiological solution supplemented with another myosin II ATPase inhibitor 2,3-butanedione monoxime (BDM) preserve cell morphology and contractile function for 2 days (15). Recently, viable cardiomyocytes were successfully isolated by enzymatic digestion of atrial tissue slices stored in cold BDM-supplemented solution (16).

Here we tested whether a similar approach, using hypothermic conditions in combination with inhibitors of myosin II ATPase, could preserve structure and function of intact heart slices prepared from the ventricle of porcine hearts. Specifically, we compare the effect of BTS and BDM, and develop and validate a method capable of extending viability and functionality of living heart slices for 6 days from slice preparation. We focus on porcine slices due to anatomical and electrophysiological similarities between pig and human hearts and therefore their relevance for translational studies.

## 2. Material and methods

### 2.1. Tissue collection

**Figure 1:**
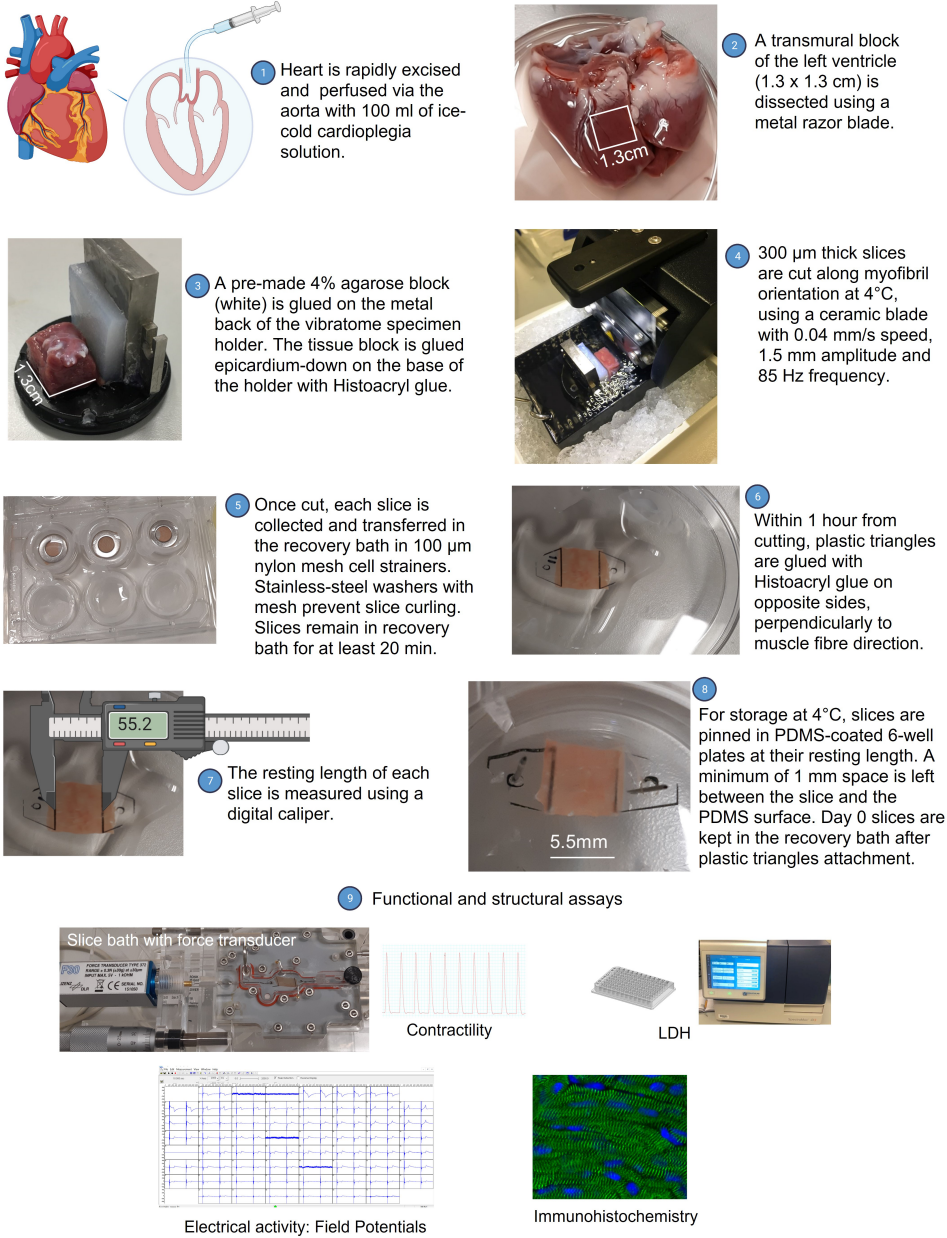
Overview of the novel method for heart slice preparation and storage. (1) After excision, the porcine heart is retrogradely perfused via the aorta with 100 ml of ice-cold cardioplegia solution. (2) Transmural tissue blocks (1.3 cm × 1.3 cm) are dissected from the left ventricle using a metal razor blade. (3) Tissue blocks are mounted on the tissue holder of a high precision vibrating microtome with epicardium down, using surgical glue Histoacryl. (4) 300 µm thick slices are prepared using a ceramic blade at a cutting speed of 0.04 mm/s in ice-cold oxygenated Tyrode's solution with 10 mM BDM. (5) Slices are incubated for at least 20 min at RT in 100 μm nylon mesh cell strainers to recover. (6) Sterile transparent plastic triangles are bond perpendicularly to muscle fibre direction on opposite side of each slice. (7) After plastic triangle attachment, the resting length of each slice is measured using a caliper. (8) For storage experiments, slices bond to plastic triangles are pinned in PDMS-coated dishes at resting length and are subsequently stored in hypothermic conditions. (9) Assays used to validate preservation of slices include contractility, LDH activity, electrical activity, and immunostaining. This figure was created using BioRender.com.

All experiments were performed under the Home Office Animals (Scientific Procedures) Act (1986) and were approved by the Animal Welfare and Ethical Review Board (AWERB) of the Pirbright Institute. Pigs six to eight weeks old (both sexes) were euthanised with an overdose of pentobarbital (Dolethal 200 mg/ml solution for injection, Vetoquinol UK Ltd, Towcester, UK) co-administered with 400 IU/kg heparin. Procedures were all performed by Personal License holders. Hearts were rapidly excised, perfused (Figure 1, step 1), and transported to the laboratory in ice-cold cardioplegia solution (NaCl 110 mM; CaCl_2_ 1.2 mM; KCl 16 mM; MgCl_2_ 16 mM; NaHCO_3_ 10 mM; pH 7.4 at 4°C) within an hour of collection.

### 2.2. Heart slice preparation and hypothermic storage

Hearts were dissected in ice-cold cardioplegia solution. Atria, right ventricle, and interventricular septum were removed. The left ventricle was cut into 1.3 × 1.3 cm^2^ blocks using a metal razor blade (Figure 1, step 2). Blocks were glued epicardium-down onto the specimen holder of a high precision vibrating microtome (Leica VT1200, UK) using Histoacryl glue (BBraun). The specimen holder was modified with a metal support and a pre-made 4% agarose block was glued between tissue and metal to avoid blade damage (Figure 1, step 3). Tissue was submerged in 4°C oxygenated (99.5% O_2_) Tyrode's solution (NaCl 140 mM; KCl 6 mM; glucose 10 mM; HEPES 10 mM; MgCl_2_ 1 mM; CaCl_2_ 1.8 mM; pH 7.4 at 4°C) containing 10 mM 2,3-butanedione monoxime (BDM). 300 μm thick slices were cut along myofibril orientation with a ceramic blade (Campden Instruments) at an advancement speed of 0.04 mm/s, amplitude of 1.5 mm and vibration frequency of 85 Hz (Figure 1, step 4), as previously described (2, 3, 11). After cutting, slices were transferred to 100 μm nylon mesh cell strainers immersed in oxygenated Tyrode's solution containing 10 mM BDM at room temperature (RT) and custom-made stainless-steel washers with mesh were placed on top of each slice to prevent curling (Figure 1, step 5). Slices were kept in cell strainers for at least 20 min to recover and warm to RT. Slices with well aligned muscle fibres were bond to small sterile plastic transparency triangles (5.5 mm wide, 0.1 mm thick) with Histoacryl glue perpendicularly to muscle fibres (Figure 1, step 6). Slices assayed on the day of preparation (day 0) were kept in nylon mesh cell strainers at RT after plastic triangles attachment for up to 7–8 h. Before storage at 4°C, the resting length of each slice was measured using a digital caliper positioned between the edge of the two plastic triangles (Figure 1, step 7). Slices were then immediately pinned at their resting length, using the tip of 21G needles, in polydimethylsiloxane-coated (PDMS, Dow Corning) 6-well plates, leaving at least 1 mm space between the slice and the PDMS surface (Figure 1, step 8). Slices were stored pinned in PDMS-coated plates containing Tyrode's solution supplemented with 10 mM BDM (pH 7.4 at 4°C) at 4°C for 1, 2, 3, and 6 days. In initial experiments, we compared slices stored in Tyrode's solution, Tyrode's solution supplemented with 10 mM BDM or Tyrode's solution supplemented with 30 µM N-benzyl-p-toluene sulphonamide (BTS) at 4°C for 1 and 2 days.

### 2.3. Viability

Slice viability was assessed using the lactate dehydrogenase (LDH) activity assay (Cytotoxicity Detection Kit LDH, Roche). Following storage at 4°C, slices were removed from PDMS-coated 6-well plates and both fresh and stored slices were washed with BDM-free oxygenated Tyrode's solution (NaCl 140 mM; KCl 4.5 mM; glucose 10 mM; HEPES 10 mM; MgCl_2_ 1 m M; CaCl_2_ 1.8 mM; pH 7.4) at RT for 15 min before transfer in 6-well plates with 2 ml prewarmed M199 medium. Plates were incubated for 2 h at 37°C in 5% CO_2_ humidified incubator (Eppendorf, CellXpert). Following incubation, culture medium was assayed for LDH activity as per the manufacturer's instructions. Briefly, 100 μl thoroughly mixed culture medium was pipetted in duplicates into 96-well plates. 100 µl of freshly prepared reaction mixture solution was added into each well and incubated for 20 min at RT. In the presence of nicotinamide adenine dinucleotide (NADH) and H^+^ from LDH activity, the catalyst (diaphorase) in the reaction mixture would reduce 2-para (iodophenyl)-3(nitrophenyl)-5(phenyl) tetrazolium chloride (INT) salts to formazan salts. The latter were detected spectrophotometrically at 492 nm absorbance using 690 nm absorbance as subtractable reference wavelength on a plate reader (Molecular Devices, Spectramax iD3). M199 medium was used as background control and was subtracted from all values, while media of slices lysed with the lysis solution provided in the kit were used as maximal LDH release controls. All measurements were presented as mean from the two duplicates and percentage of maximal LDH release controls, normalised to slice weight (in mg). Plastic transparency triangles were gently detached, slices were blotted dry and weighted on a precision balance after the 2 h incubation in M199 medium to determine slice weight.

### 2.4. Contractility

Slices were washed in BDM-free oxygenated Tyrode's solution at RT for 30 min, followed by 10 min wash at 37°C, before transfer in a horizontal organ bath [Mayflower, Hugo Sachs Elektronik (HSE), Germany] equipped with an F30 isometric force transducer (HSE) (Figure 1, step 9). Slices were attached with minimum preload to the force transducer hook and to the fixed hook via plastic transparency triangles and were submerged in 4 ml prewarmed (37°C) BDM-free oxygenated Tyrode's solution. Slices were continuously superfused with 37°C BDM-free oxygenated Tyrode's solution and field stimulated at 1 Hz pacing frequency, with a pulse of 5 ms duration and 10 V amplitude, using a pair of platinum electrodes placed on opposite sides of the bath and connected to a Grass S88 stimulator (Natus Medical Inc, Pleasanton, USA). To determine maximum contractility slices were stretched stepwise by 5% every 2 min, until isometric contraction reached the maximum before clearly declining. Contractility traces were recorded using PowerLab (AD Instruments, 8/35) and analysed for maximal amplitude, time to peak contraction and time to 100% relaxation using LabChart v7.3.8 software (AD Instruments). Amplitude was converted to force (mN), using a scale factor of 10 (measured during force transducer calibration). Force was normalised to the cross-sectional area of the heart slice (mN/mm^2^).

### 2.5. Field potential recordings

For field potential recordings, slices were positioned in glass multi-electrode array (MEA) plates, held in contact with the electrodes by a slice holder, and continuously superfused with BDM-free oxygenated Tyrode's solution at 37°C. The MEA plates (MEA2100, Multi Channel Systems, Germany) contained 60 microelectrodes arranged in an 8 × 8 matrix, with 100 μm electrode diameter and an inter-electrode distance of 700 μm, providing a recording area of about 5 × 5 mm^2^ in the centre of the MEA plate. Slices were electrically stimulated via one of the MEA microelectrodes using bipolar pulses of 500 μs duration, 1–2 V amplitude and 1 Hz or 2 Hz frequency. After a 15 min equilibration period, field potentials were recorded simultaneously from all 60 microelectrodes with a sampling frequency of 50 kHz. Field potential duration (FPD), defined as the time between the depolarisation peak (minimum peak) and the repolarisation peak (the peak of the second wave form, akin to the T wave of an electrocardiogram), was analysed using Clampfit software (Axon Instruments, USA). Recordings from all microelectrodes were averaged to obtain the FPD of each slice.

### 2.6. Immunohistochemistry and confocal microscopy

Freshly prepared slices and slices stored at 4°C in BDM-supplemented Tyrode's solution were washed in phosphate buffered saline (PBS) and fixed in 4% paraformaldehyde for 15 min at RT. Slices were washed in PBS 4–5 times (10–15 min each) on a shaker at RT, before permeabilization and blocking in 1% Triton X-100% and 1% bovine serum albumin for 2 h at RT. Slices were incubated with primary antibodies mouse monoclonal anti-α-actinin (1:100; clone EA-53, Sigma-Aldrich) or mouse monoclonal anti-vimentin (1:1,000; clone V9, Sigma-Aldrich) for 2 days at 4°C. Slices were washed in PBS 3 times (30 min each) and incubated with secondary antibody donkey anti-mouse Alexa488 (1:400; 2 h RT). After secondary antibody, slices were washed in PBS and nuclei were stained with DAPI (1:1,000; 10 min at RT). After a final wash in PBS, slices were stored in PBS at 4°C until imaging. Images were taken with a Nikon Ti-Eclipse A1M confocal microscope using a 10x-objective for vimentin and a 40x-objective for α-actinin. For sarcomere length quantification, the distance between >10 consecutive sarcomere was measured using the ImageJ intensity profile plugin along a line perpendicular to the visible α-actinin striation pattern (Figure 6). A minimum of 15 cells per slice were analysed and the average of the cells was used as slice sarcomere length. Stromal cells content was quantified as percentage area of vimentin per total tissue area. A minimum of 10 areas were analysed per slice and averaged to provide one single value.

### 2.7. Data analysis

One-way ANOVA was used to compare measurements from different groups, with significant group difference compared using Tukey's multiple comparison post-hoc test. Statistical analysis was performed using GraphPad Prism version 8.4.3 for Windows (GraphPad Software, San Diego, California USA, www.graphpad.com). Data are presented as mean ± SEM, and a *p* value of <0.05 was considered significant.

## 3. Results

### 3.1. Contractility and viability

**Figure 2:**
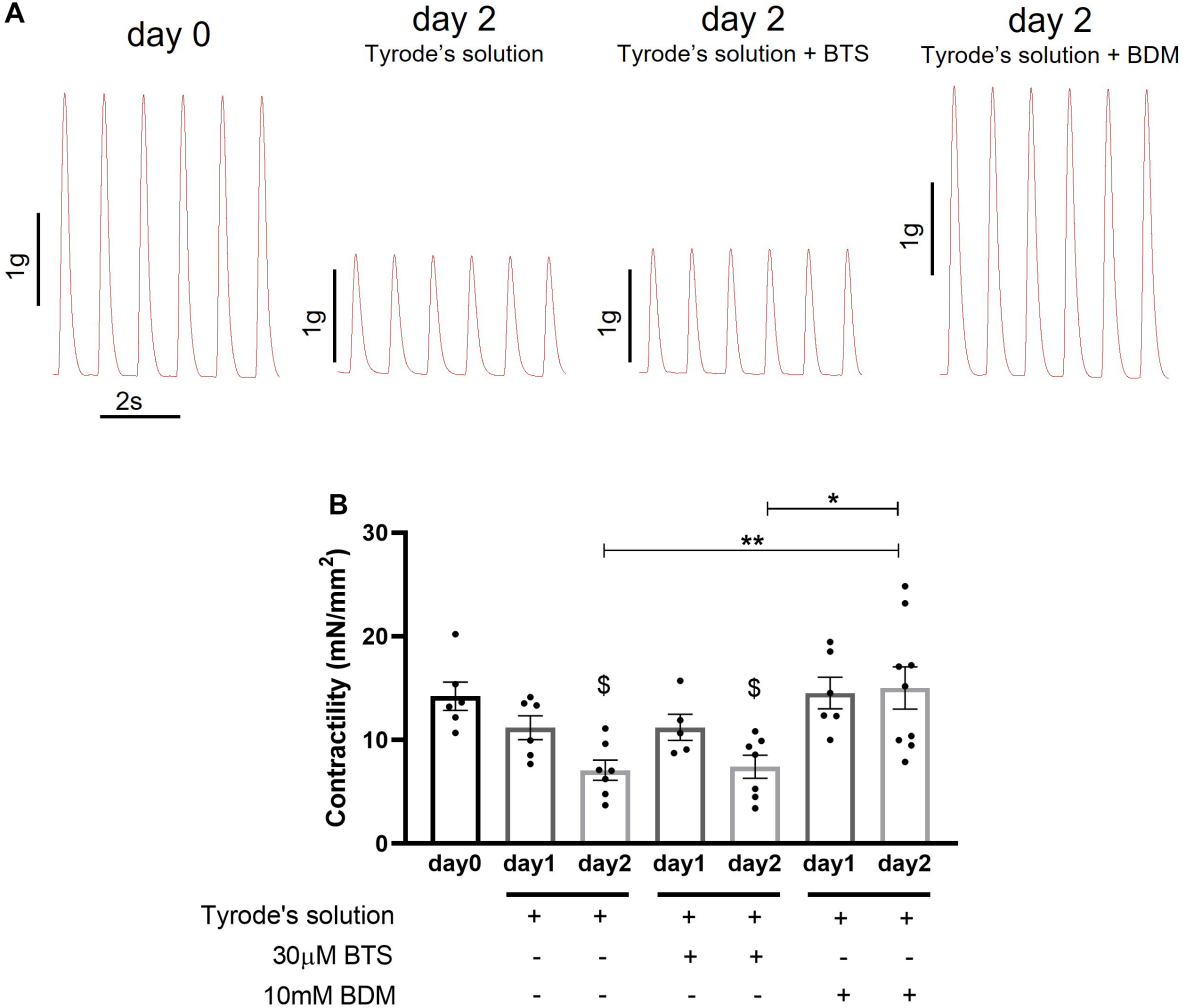
Contractility of porcine heart slices stored at 4°C in Tyrode's solution, Tyrode's solution supplemented with BTS and Tyrode's solution supplemented with BDM. (**A**) Representative contraction traces from freshly prepared porcine slices (day 0) and porcine slices stored for 2 days at 4°C in the 3 different solutions. (**B**) Contractility of porcine slices over the 2-days storage period in the 3 solutions. Slices were field stimulated at 1 Hz pacing frequency. Bars represent means ± SEM. Black dots represent individual data points (*n* = 5–9 slices/3–4 hearts). $ *p* < 0.05 compared to day 0; *p < 0.05, **p < 0.01 between two groups (one-way ANOVA with Tukey's multiple comparisons test).

To optimise a preservation protocol that maintains viability and function of heart slices, we initially tested storage of slices in 3 hypothermic (4°C) solutions; (i) Tyrode's solution, (ii) Tyrode's solution supplemented with 10 mM BDM and (iii) Tyrode's solution supplemented with 30 µM BTS. Maximum contractility (maximum force generated at 1 Hz stimulation normalised to slice cross-sectional area) was measured using a force transducer after 1 day and 2 days of storage. Freshly prepared slices (day 0) were used as control. Slices were washed in Tyrode's solution for a minimum of 40 min to remove BDM and BTS before transfer in the horizontal organ bath where they were superfused at 37°C for further 10 min prior to contractility measurements. As shown in Figure 2, all solutions were able to preserve contractility of slices for 1 day, however after 2 days only slices stored in BDM-supplemented Tyrode's solution maintained contractility similar to fresh slices (day 0). Slices stored for 2 days in Tyrode's solution or BTS-supplemented Tyrode's solution showed significant reduction in contractility compared to day 0. Significant differences in contractility were also observed between slices stored for 2 days in Tyrode's solution or BTS-supplemented Tyrode's solution compared to BDM-supplemented Tyrode's solution.

**Figure 3:**
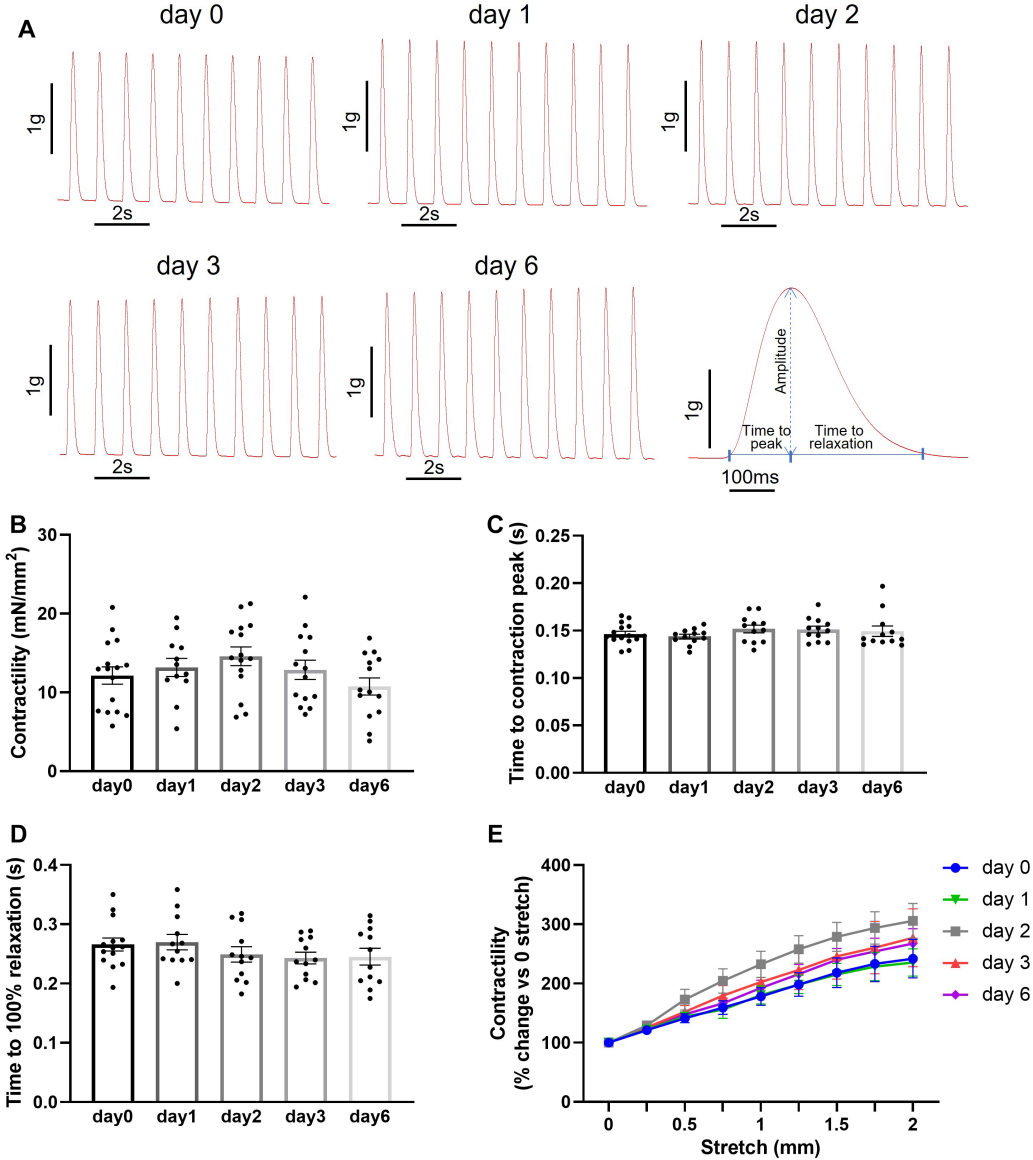
Contractile function of porcine heart slices after storage at 4°C in Tyrode's solution supplemented with BDM over a 6-day period. (**A**) Representative contraction traces from freshly prepared porcine slices (day 0) and porcine slices after storage at 4°C in BDM-supplemented Tyrode's solution for 1, 2, 3 and 6 days. Right: contraction trace showing parameters analyzed. (**B**) Contractility of porcine slices over storage time. (**C,D**) Time to contraction peak and time to 100% relaxation. Bars represent means ± SEM. Black dots represent individual data points (*n* = 12–16 slices/4 hearts). (**E**) Contractility produced by porcine slices under stepwise stretch and 1 Hz stimulation (*n* = 16 slices/4 hearts at day 0; *n* = 12 slices/4 hearts at day 1, 2, 3 and 6; means ± SEM).

**Figure 4:**
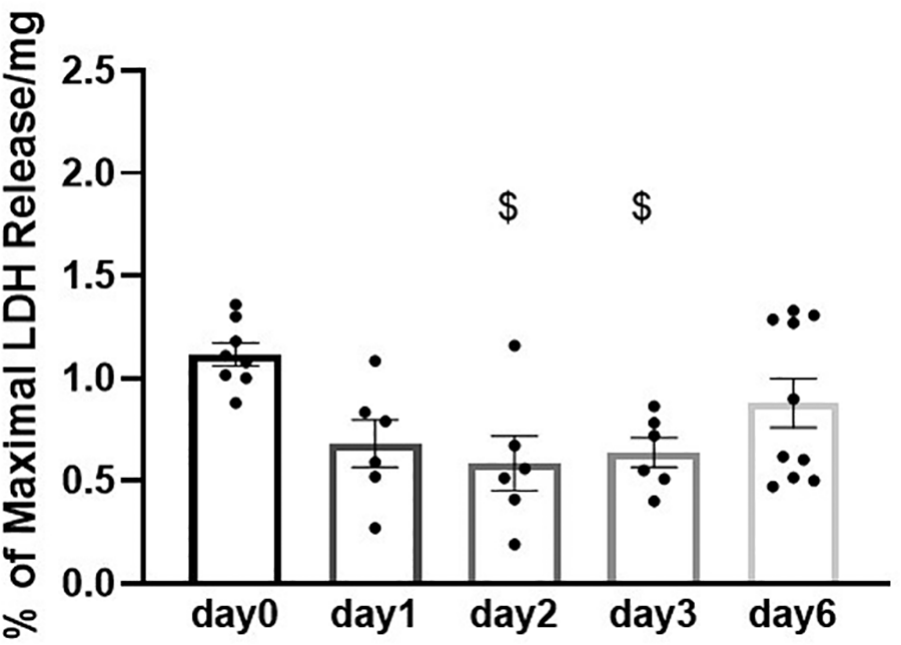
Viability of porcine heart slices stored at 4°C in BDM-supplemented Tyrode's solution over 6 days. LDH release in the media is expressed as percentage (%) of positive control (LDH levels in media from slices lysed with the lysis solution) normalized to slice weight (in mg). Bars represent means ± SEM. Black dots represent individual data points (*n* = 6–10 slices/3–4 hearts). $ *p* < 0.05 compared to day 0 (one-way ANOVA with Tukey's multiple comparisons test).

We then assessed the feasibility of preserving slices in BDM-supplemented Tyrode's solution at 4°C for longer than 2 days. Maximum contractility was measured after 1, 2, 3 and 6 days of storage. No statistically significant differences were observed in the contractility of slices stored for up to 6 days in these conditions (Figures 3A,B). Similarly, no statistically significant differences were observed in time to contraction peak and time to relaxation (Figures 3D). Furthermore, stretch-induced increase in contractility was comparable in all slices over the 6 days storage period (Figure 3E). Slice viability was further assessed by quantification of LDH activity. LDH levels did not increase with time in storage indicating cell integrity is maintained in slices over the assessed period (Figure 4). A significant decrease in LDH activity was observed in slices after 2–3 days in hypothermic storage with BDM-supplemented solution compared to fresh slices (day 0) suggesting some recovery from the initial cutting insult occurred during the first few days after slice preparation (Figure 4).

### 3.2. Field potential duration

**Figure 5:**
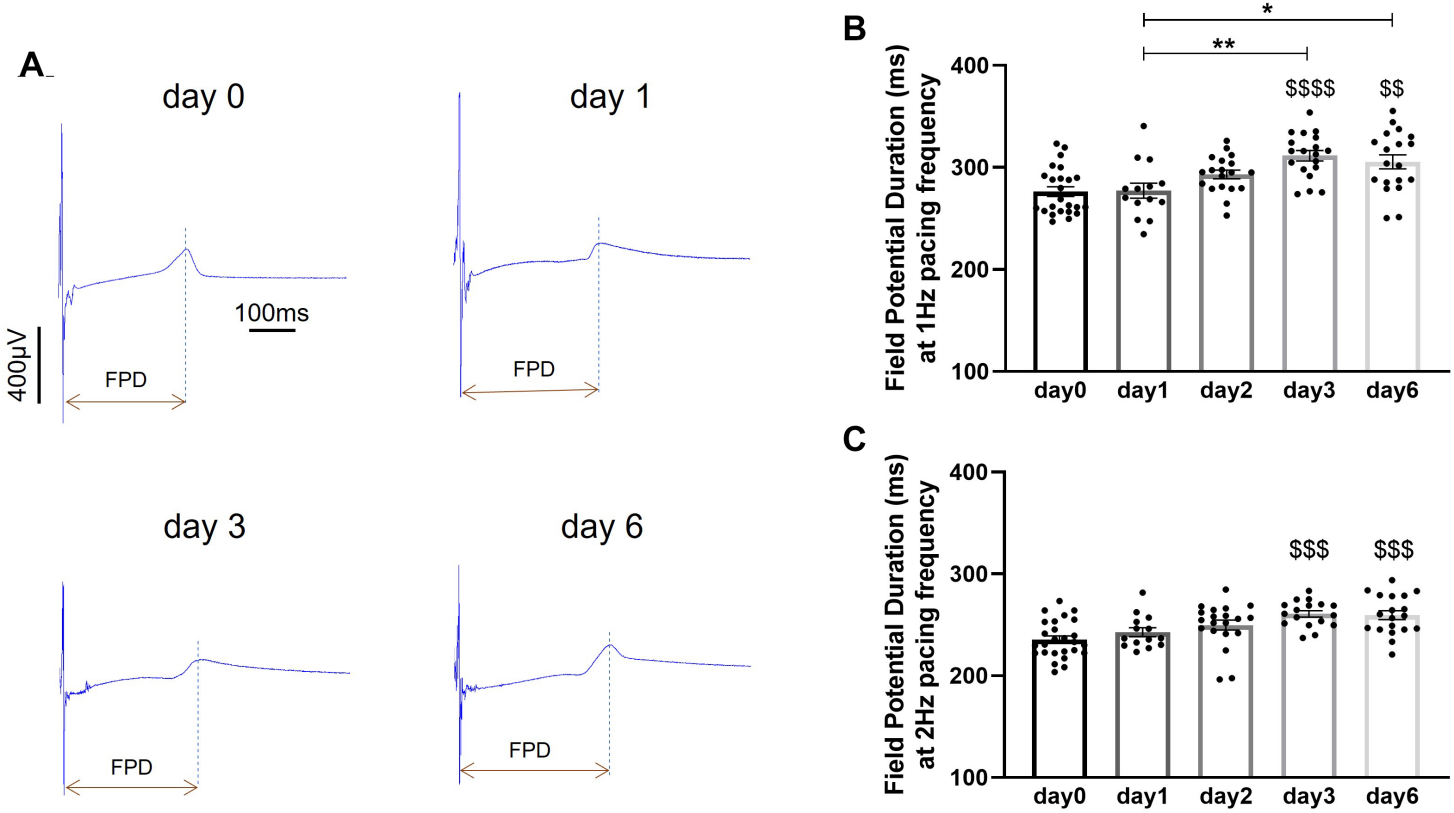
Field potential duration in porcine heart slices stored at 4°C in BDM-supplemented Tyrode's solution over 6 days. (**A**) Representative field potential traces recorded during point stimulation at 1 Hz frequency in freshly prepared porcine slices (day 0) and porcine slices stored at 4°C in BDM-supplemented Tyrode's solution for 1, 3 and 6 days. FPD: field potential duration. (**B,C**) Field potential duration at 1 Hz and 2 Hz pacing frequency. Bars represent means ± SEM. Black dots represent individual data points (*n* = 14–24 slices/4 hearts). $$ *p* < 0.01, $$$ *p* < 0.001, $$$$ *p* < 0.0001 compared to day 0; *p < 0.05, **p < 0.01 between two groups (one-way ANOVA with Tukey's multiple comparisons test).

Preservation of electrical activity in porcine heart slices stored at 4°C in BDM-supplemented Tyrode's solution was assessed by measuring field potentials over 60 electrodes covering the entire slice surface. Field potentials, recorded during electrical pacing at 1 Hz and 2 Hz frequency, were used to measure field potential duration (FPD). Field potentials were consistently detected at all 60 electrodes in slices stored for up to 6 days. No significant changes in FPD were observed in slices stored for 1 day and 2 days in BDM-supplemented Tyrode's solution at 4°C compared to control values obtained in fresh slices (day 0), however FPD significantly increased after 3 days and 6 days in this storage conditions (Figure 5). FPD recorded at 1 Hz pacing frequency increased by 12.7 ± 1.8% and 10.5 ± 2.5% after 3 days and 6 days of storage respectively. At 2 Hz pacing frequency, FPD increased by 10.7 ± 1.3% after 3 days and by 10.2 ± 1.9% after 6 days of storage. FPD was dependent on stimulation frequency and was significantly shorter at 2 Hz vs. 1 Hz at all time points (day 0: *p* < 0.0001; day 1: *p* < 0.001; day 2: *p* < 0.0001; day 3: *p* < 0.0001; day 6: *p* < 0.0001).

### 3.3. Structural properties: sarcomere length and stromal cells

**Figure 6:**
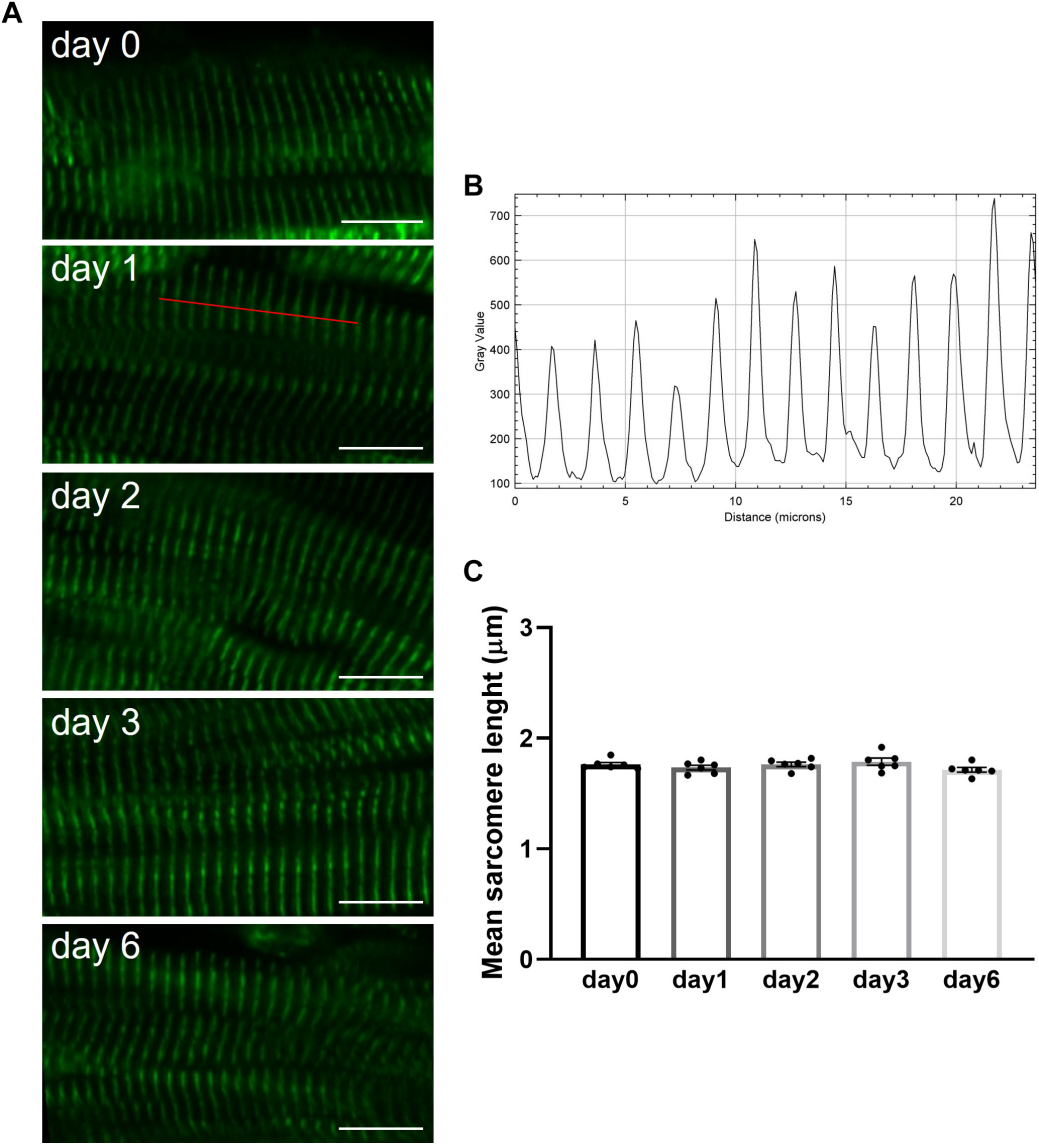
Sarcomere length in porcine heart slices is preserved over 6 days during storage at 4°C in BDM-supplemented Tyrode's solution. (**A**) Confocal microscopy images of slices stained for α-actinin briefly after preparation (day 0) or after storage at 4°C in BDM-supplemented Tyrode's solution for 1, 2, 3 and 6 days. Scale bar: 10 µm. (**B**) Intensity profile of the user-drawn path (red line in day 1 image) used to quantify sarcomere length. (**C**) Mean sarcomere length of fresh slices (day 0) and slices stored at 4°C in BDM-supplemented Tyrode's solution for different duration. Bars represent means ± SEM. Black dots represent individual data points (*n* = 6 slices/3 hearts; *p* > 0.05; one-way ANOVA with Tukey's multiple comparisons test).

**Figure 7:**
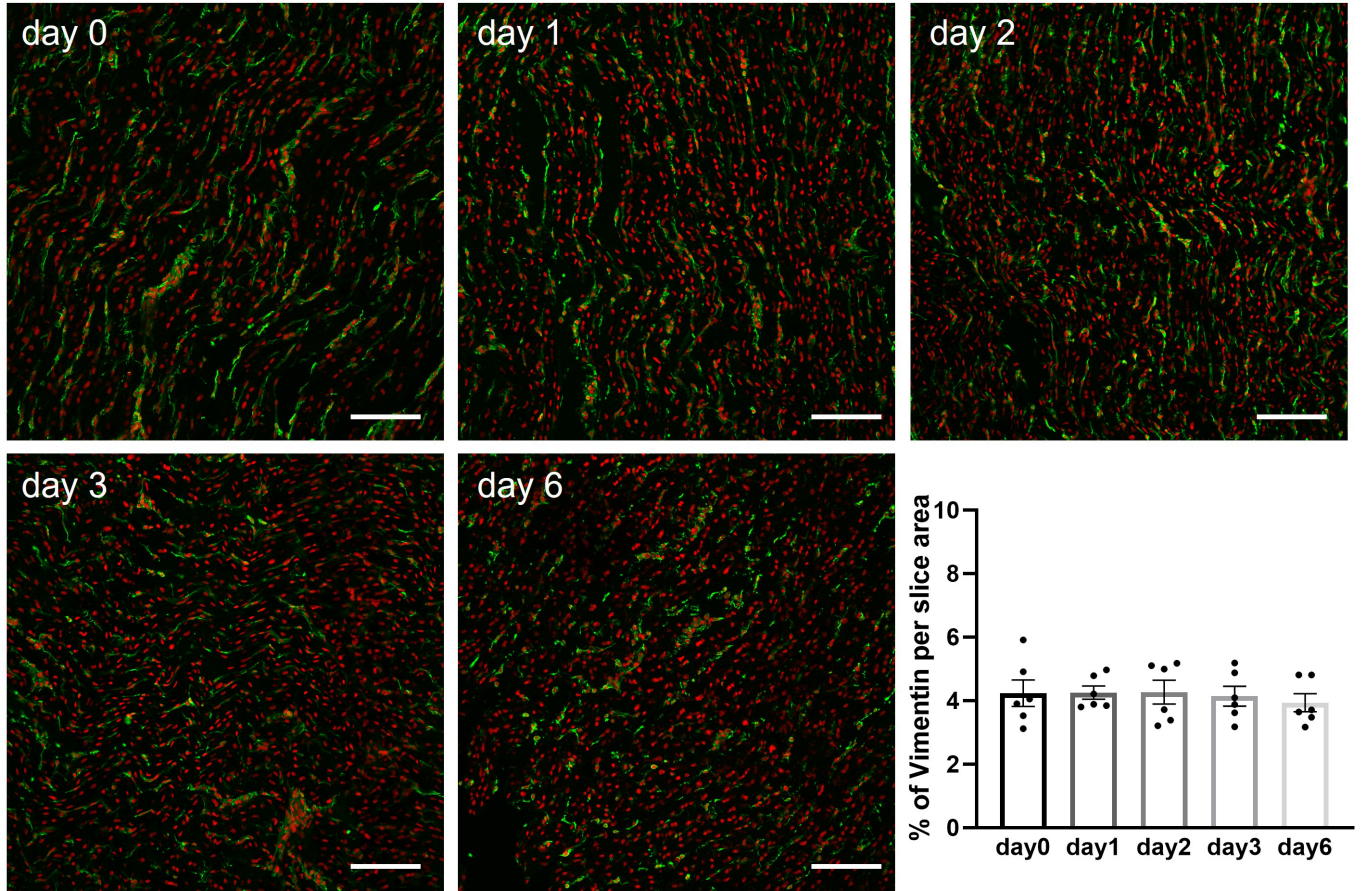
Quantification of stromal cells in porcine heart slices stored at 4°C in BDM-supplemented Tyrode's solution over a 6-day period. Representative confocal images of slices stained for vimentin (green) and nuclei (red). Scale bars: 100 µm. Bar graph summarizing quantification of vimentin in slices stored for different duration. Bars represent means ± SEM. Black dots represent individual data points (*n* = 6 slices/3 hearts; *p* > 0.05; one-way ANOVA with Tukey's multiple comparisons test).

Briefly after preparation or after hypothermic storage in BDM-supplemented Tyrode's solution, slices were fixed and processed for immunohistochemical staining as previously described (17). Confocal images of intact slices stained for *α*-actinin revealed the presence of a regular z-disc pattern in freshly prepared porcine slices (day 0) as well as in slices stored over the 6-day period (Figure 6A). Quantitative analysis of confocal images demonstrated that sarcomere length remained constant in slices during hypothermic storage in the presence of BDM (Figures 6B,C). Staining of slices with vimentin further demonstrated that stromal cells remained quiescent during storage at 4°C in BDM-supplemented Tyrode's solution as no significant differences were observed in the percentage of vimentin stain per tissue area between fresh slices (day 0) and slices stored for 1, 2, 3 or 6 days (Figure 7).

## 4. Discussion

Here we present a method to extend viability and functionality of living heart slices for several days after slice preparation. We demonstrate that storing porcine heart slices at 4°C in Tyrode's solution supplemented with 10 mM BDM preserves slice viability, contractility and morphology for up to 6 days. Furthermore, we show that electrophysiological function, assessed by measuring extracellular field potentials, remains stable for up to 2 days, followed by a slight but significant prolongation in FPD after 3–6 days. These results suggest that slices prepared from the same heart can be used for assays over several consecutive days, leading to a significant reduction in the number of animals needed for basic cardiac research. As >20 slices can be prepared from the heart of small rodents, and >100 slices from a single porcine or human heart, extending viability and functionality of heart slices can maximize experimental output and minimize tissue waste. It also allows more experimental interventions to be evaluated in the same heart to gain more in-depth understanding of the research question. Furthermore, a robust and simple method to preserve heart slices in hypothermic conditions can promote sharing of living slices between laboratories, facilitating collaborations and accelerating both basic and translational research.

Building on previous work showing that single cardiomyocytes maintain morphology and contractile function for days when stored at low temperature in the presence of myosin ATPase inhibitors BTS and BDM (14, 15), we initially stored porcine heart slices at 4°C for 2 days in Tyrode's solution supplemented with 30 µM BTS, 10 mM BDM or in the absence of myosin ATPase inhibitors. Our results show that BDM preserved slice contractility over the 2-day storage period, however contractility was significantly reduced in slices stored with BTS similarly to slices stored in the absence of myosin ATPase inhibitors. The difference between BTS and BDM results could be due to the different specificity and concentration of the two compounds. BTS is a specific inhibitor of skeletal myosin ATPase, known to have little effect on cardiac muscle (18). This specificity could limit the ability of BTS to suppress slice contraction during cold storage, which is required to prolong the viability of heart slices. Further, the 30 µM BTS concentration used in our study, which was selected for its ability to promote survival of canine cardiomyocytes (14), may be too low to achieve sufficient inhibition of cardiac myosin ATPase in heart slices. Mechanisms independent from myosin ATPase inhibition cannot however be excluded as BDM has been reported to exert multiple non-specific effects, including inhibition of several ion channels (19, 20) and dephosphorylation of membrane phosphoproteins (21, 22). Blebbistatin, a more specific cardiac myosin ATPase inhibitor, was not used in this study as blebbistatin was previously reported to alter cardiomyocyte contractility and calcium handling within 24 h in cold storage (15).

Storing slices at 4°C in BDM-supplemented Tyrode's solution maintained contractility, viability, and structure comparable to fresh slices for up to 6 days after slice preparation. BDM has previously been shown to improve survival of cardiomyocytes in cold storage by reducing contractile force and metabolic demand (15, 23). BDM coupled with low temperature also improves viability of heart slices by preventing tissue contraction during the slicing procedure. After preparation, slices are maintained in BDM at room temperature for recovery. In these conditions slices have been shown to maintain stable electrophysiological characteristics for 12 h (2), however when temperature was reduced to 12°C this window was successfully prolonged to 28 h (13). In this study we did not test whether storing slices in BDM at RT or physiological temperature is sufficient to preserve slice viability and functionality for days, however the above studies together with evidence that inhibition of the myosin ATPase at RT or 37°C is unable to preserve viability of isolated cardiomyocyte (14) suggest that low temperature is an essential parameter for optimal preservation of heart slices. We limited the concentration of BDM to 10 mM in our study as this concentration is sufficient to inhibit tissue contractility during cutting and slice recovery (2), and its effect has been shown to be rapidly reversible upon washout (11, 24–26).

Crucially, storing slices in Tyrode's solution containing 10 mM BDM at 4°C preserved contractile function, including contractile force and contraction kinetics, and did not increase LDH activity for at least 6 days after slice preparation. All slices responded to field stimulation and stepwise stretch with increased force generation to a maximum value of 11 ± 1 mN/mm^2^ after 6 days storage, similar to the maximum force measured in fresh porcine slices in this study (12 ± 1 mN/mm^2^), and fresh porcine, rabbit and human slices in previous work (11, 27, 28). Importantly, slice-to-slice variability in contractility, which is due to epicardial-endocardial differences, remained constant over storage time, indicating that intrinsic transmural heterogeneities in mechanical properties are well conserved in heart slices for several days after preparation. To limit the effect of slice-to-slice variability during experimental applications, cumulative interventions on the same slice or pairing consecutive slices would avoid the limitation of transmural heterogeneities. On the other hand, however, the ability to study specific regions of the myocardium is an important advantage of heart slices, therefore storing and treating epicardial, midmyocardial and endocardial slices separately could provide important insights into regional differences of healthy and disease hearts.

Electrophysiological function, assessed as FPD, remained stable for up to 2 days when slices were stored in Tyrode's solution supplemented with 10 mM BDM at 4°C. Interestingly, we observed that FPD was prolonged in slices after 3 days and 6 days storage compared to freshly prepared slices. A similar prolongation in action potential duration (APD) (∼7%) was previously reported in guinea pig heart slices after 24 h storage at 12°C in high potassium Tyrode's solution supplemented with BDM (13). The reasons for this FPD/APD prolongation remain unclear and warrant further investigation. Incomplete reversibility of the effect of BDM following long-term exposure or remodeling of repolarizing ion channels during storage could explain these results.

In terms of structure, our work focused on *α*-actinin organization, sarcomere length, and stromal cells. Both sarcomere length and content of stromal cells remained unchanged over the 6 days storage period in BDM. This contributes critically to the well-preserved slice contractility, an output of the integrity of the contractile apparatus consisting of multiple structural components with intact energy production and metabolic function. Average sarcomere length measured in porcine slices following fixation ranged from 1.79 to 1.71 µm. These values are similar to those we reported for freshly prepared rat slices (1.79 µm) imaged live prior to fixation (29). Combination of BDM and cold storage is likely a key factor in the preservation of sarcomere length in our porcine slices, in agreement with previous results reported for isolated cardiomyocytes (15). No increase in the number of cells expressing vimentin was observed for up to 6 days after slice preparation indicating absence of stromal cells proliferation. This is in stark contrast with the significant increase in stromal cells, fibrosis, and enlarged extracellular spaces reported in heart slices during culture (24, 30). Standard tissue culture conditions are likely to induce cell dedifferentiation, proliferation, and phenotypic changes and hence a deviation from the property of the native tissue. During culture, mechanical stimulation has been shown to reduce cell dedifferentiation and promote tissue preservation (27), in contrast with hypothermic storage where inhibition of mechanical contraction is critical to promote cells and tissue preservation. Furthermore, while application of preload was necessary to maintain structural and functional properties of slices in culture (27, 28), no preload was needed to preserve slices in hypodermic conditions under mechanical uncoupling. Future work assessing gap junctions and conduction properties of slices will be needed to further validate the preservation of intact cell-cell connections during long-term storage. Analysis of calcium cycling, together with comprehensive genes and proteins quantification will be also important to fully validate slice preservation.

In summary, our data show that BDM can be used in combination with hypothermic conditions to preserve morphology, viability, and contractility of porcine heart slices for 6 days. This significantly extends the period over which acute heart slices can be used for functional studies. The simplicity of the method allows for rapid adoption by other laboratories without the need for complex and specialized equipment which is required to extend the viability of slices in culture conditions (27, 31). Wide adoption and application of the method to rodent models and human tissue, in addition to large animal models, has the potential to significantly lower experimental costs and reduce the number of animals needed for cardiac research.

## Data Availability

The original contributions presented in the study are included in the article/Supplementary Material, further inquiries can be directed to the corresponding author.
